# Induced Voltage Linear Extraction Method Using an Active Kelvin Bridge for Disturbing Force Self-Sensing

**DOI:** 10.3390/s16050739

**Published:** 2016-05-20

**Authors:** Yuanyuan Yang, Lei Wang, Jiubin Tan, Bo Zhao

**Affiliations:** Harbin Institute of Technology, D-401 Science Park, No. 2 Yikuang Street, Harbin 150080, China; yyy42616500@126.com (Y.Y.); jbtan@hit.edu.cn (J.T.); hitzhaobo@hit.edu.cn (B.Z.)

**Keywords:** giant magnetostrictive actuator, self-sensing, Kelvin bridge, induced voltage

## Abstract

This paper presents an induced voltage linear extraction method for disturbing force self-sensing in the application of giant magnetostrictive actuators (GMAs). In this method, a Kelvin bridge combined with an active device is constructed instead of a conventional Wheatstone bridge for extraction of the induced voltage, and an additional GMA is adopted as a reference actuator in the self-sensing circuit in order to balance the circuit bridge. The linear fitting of the measurement data is done according to the linear relationship between the disturbing forces and the integral of the induced voltage. The experimental results confirm the good performance of the proposed method, and the self-sensitivity of the disturbing forces is better than 2.0 (mV·s)/N.

## 1. Introduction

Giant magnetostrictive actuators (GMAs) are a new type of micro-displacement actuator that exhibit good driving characteristics under the action of a constant force [1,2,3]. Their power output is sufficiently large under low- or high-frequency, and their strain output is sufficiently large under low magnetic field drive. GMAs have therefore been widely used in wideband vibrators, medical equipment and ultrasonic transducers, *etc.* [4,5,6,7].

The concept of self-sensing actuator was described by Jeffrey J. Dosch *et al.* in 1992 for a piezoelectric actuator [8]. Then, the application research on the vibration suppression was conducted in the same year [9]. Design of a self-sensing magnetostrictive actuator was firstly proposed by Jon Pratt [10,11]. Giant magnetostrictive materials (GMMs) with the inverse magnetostrictive effect (Villari effect) can detect the displacement [12,13], velocity [14], stress and forces [15,16] without the need for additional sensors, thus simplifying the system structure. GMMs are therefore used for simultaneous realization of self-sensing and precision positioning.

However, nonlinear displacement output appears owing to the coupling between the positive magnetostrictive effect and the inverse magnetostrictive effect. Another drawback in the realization of positioning precision is the nonlinear method for the extraction of the induced voltage.

To enhance the positioning precision of GMAs, the dynamic measurement of the disturbing forces is modified into measurement of the electrical and magnetic signals caused by the disturbing forces. The induced voltage in the drive coil of GMA is regarded as the indirect measurement signal. Depending on the inverse magnetostrictive effect, strain appears in a GMM rod when an external force acts on it. The magnetization of the GMM rod will then change, and the magnetic flux density within the material changes accordingly. Finally, the series of changes is reflected in the induced voltage signal. In other words, GMA self-sensing of external disturbing forces is achieved by the indirect measurement of the induced voltage signal.

An equivalent circuit model of the GMA was proposed by Jon Pratt and Alison B. Flatau in 1993 [10,11], and the model indicated that the self-sensing actuator usually works in the linear region under the conditions of adequate pre-bias magnetic field and preloading stress. The GMA self-sensing signal detection method based on a Wheatstone bridge was then proposed. In 1998, from Germany Saarland university LPA laboratory, Bernd Clephas and Hartmut Janocha proposed GMA self-sensing by evaluating the inductivity of the exciting coil [17]. In 2004, Kuhnen K. *et al.* compared the two kinds of self-sensing methods proposed by the LPA, and found that the coil inductance indirect measurement method is easily affected by the irreversible current and the method using hall sensors to detect the magnetic field has good linearity [18]. In 2008, Hui Duan *et al.* applied the Jiles-Atherton (J-A) non-linear GMA model to the detection bridge circuit and added an observer in the control model simultaneously. Their experimental results were satisfactory for practical applications [14]. In 2009, Wang Xinhua analyzed the balance bridge inductance by using the least means square self-adapting algorithm in order to reduce signal distortion [19].

The application of GMAs as bidirectional transducers with self-sensing properties is the development direction of micro displacement actuators, and the self-sensing performance is the key factor restricting this application. The conventional method for extracting induced voltage with the Wheatstone bridge [10,14,19] is weak nonlinear, and the high frequency information is lost. Furthermore, according to the inverse magnetostrictive effect and the GMA equivalent circuit model, the value of the disturbing forces is directly proportional to the integral of the induced voltage rather than itself. The above two facts considerably affect the improvement of the measurement precision and sensitivity.

In this paper, we proposed a linear extraction method for induced voltage based on the Kelvin bridge. A reference GMA was applied to the bridge circuit, and the Kelvin bridge combined with an active device was configured to solve nonlinearity problems in GMA self-sensing. The active device, a kind of high precision and zero-drift amplifier, was chosen as a voltage-follower. The active Kelvin bridge had strong common-mode rejection ability due to that the input impedance of the amplifier was greater than the impedance of GMA. So, the induced voltage was extracted with no distortion and no loss of the high frequency information. Then, linear extraction of the induced voltage can be realized and the disturbing forces can be detected over a broad band. The proposed method has been used in related research on a micro displacement actuator, especially on a differential type positioning device on account of two GMAs working at the same time. The positioning performance was improved when the friction influence on load was offset by applying the method.

## 2. Magnetostrictive Self-Sensing Mechanism Analysis

GMA exhibits two-way switch features owing to the co-existence of the positive magnetostrictive effect and the inverse magnetostrictive effect. When the GMA simultaneously functions as an actuator and a sensor element, its signal and energy transfer process are clearly described as a double-terminal network [20], and the energy transfer is an electrical magnetic mechanical mutual conversion process.

**Figure 1:**
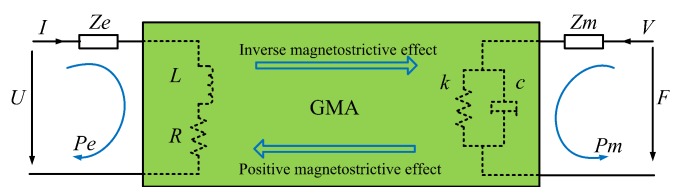
Double-terminal network energy transfer model of a giant magnetostrictive actuator (GMA).

Depending on the positive magnetostrictive effect, the GMA equivalent electrical impedance is $Z_{e}$ when it works as an actuator. In other words, the inputs are voltage *U* or current *I* and the outputs are mechanical parameters, such as force or displacement. Similarly, depending on the inverse magnetostrictive effect, the GMA mechanical equivalent impedance is $Z_{m}$ when it works as a sensor, and the inputs are force *F* or speed *V* and the outputs are electrical parameters, such as current or voltage. The signal conversion is accompanied by energy conversion, as shown in Figure 1. The impedance equation corresponding to mechanical-electrical double-terminal network model can be described as

(1) $$\left\{\begin{array}{c} U=Z_{e}I+T_{em}V\\ F=T_{me}I+Z_{m}V \end{array}\right.$$

where $T_{em}$ and $T_{me}$ are the mechano-electrical and electro-mechanical conversion coefficients, respectively. All physical quantities in Equation (1) are Laplace expressions. This equation shows that a strong magneto-mechanical coupling effect exists between the electrical and mechanical quantities owing to the co-existence of the positive and inverse magnetostrictive effects.

The research results of Jon Pratt and Alison B. Flatau have shown that the basis of the self-sensing GMA design is the linear magnetoelastic material model when the actuators function under appropriate pre-bias magnetic field and preloading stress [10]. For a giant magnetostrictive rod with one end fixed and the other end free to expand under the conditions of constant temperature, small enough input signal, and closed magnetic circuit, the GMM magneto-mechanical coupling relationship is expressed by the first kind of linear piezomagnetic equation [20], as given by Equations (2) and (3):

(2) $$\varepsilon =\frac{\sigma }{E}+d_{33}H$$

(3) $$B=d_{33}^{\prime }\sigma +\mu^{\sigma }H$$

where *ε* is strain, *σ* is the GMM rod stress, *E* is the Young’s modulus, $d_{33}$ is the magneto-mechanical coupling coefficient, *H* is the driven magnetic field intensity, *B* is the magnetic induction intensity, and $\mu^{\sigma }$ is the permeability of materials under a certain stress. It is worth noting that $d_{33}$ of Equation (2) is the differential of the magnetic field relative to the strain, *i.e.*, the magnetostrictive coefficient. Further, $d_{33}^{\prime }$ of Equation (3) represents the differential of the magnetic induction intensity relative stress, *i.e.*, the inverse magnetostrictive coefficient. When the GMA functions under a sufficiently small signal drive, the above two are deemed to be approximately equal [20]. Based on the linear piezomagnetic Equations (2) and (3), the magnetic field intensity *H* inside the coil is equal to the product of the number of coil turns per unit length $n=N/l_{coil}$ and the drive current *i*. Then, the magnet flux ϕ flowing from the GMM rod cross section is

(4) $$\phi =A_{coil}d_{33}\sigma +A_{coil}\mu^{\sigma }ni$$

where $l_{coil}$ is the length of the coil, *N* is the number of coil turns, $A_{coil}$ is cross-sectional area of the excitation coil. Then, the induced voltage $u_{m}\left(t\right)$ of the coil is described by the following equation

(5) $$u_{m}\left(t\right)=N\frac{d\phi }{dt}=NA_{coil}d_{33}\frac{d\sigma }{dt}+L_{d}\frac{di}{dt}$$

where $L_{d}$ is self-inductance coefficient.

(6) $$L_{d}=\mu^{\sigma }N^{2}\frac{A_{coil}}{l_{coil}}$$

According to the relationships among stress, strain, and modulus of elasticity, the functional relationship between the load disturbance force $F\left(t\right)$ and the coil induced voltage $u_{m}\left(t\right)$ is shown in Equation (7).

(7) $$F\left(t\right)=A_{r}\int d\sigma =\frac{A_{r}}{NA_{coil}d_{33}}\left(\int u_{m}dt-L_{d}i\right)$$

where $A_{r}$ is the cross-sectional area of the GMM rod. In conclusion, the external disturbing force F(t) can be deduced by the induced voltage $u_{m}\left(t\right)$ and an equivalent self-induction $L_{d}$ for the linear piezomagnetic equation of the inverse magnetostrictive effect. Thus, the measurement of the external disturbing force has been converted to the measurement of $u_{m}\left(t\right)$ and i(t).

## 3. Sensing Signal Separation Method Based on the Kelvin Bridge

**Figure 2:**
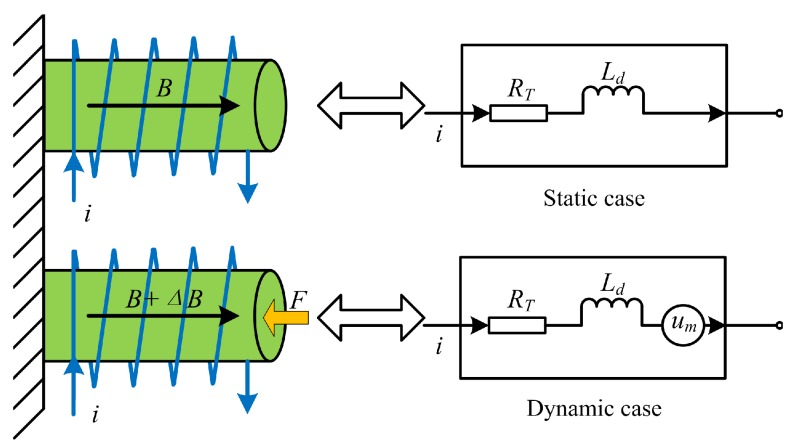
Equivalent impedance of GMA in the static case and the dynamic case.

The equivalent impedance of an actuator influenced by the external disturbing force is illustrated in Figure 2. In the static case, no external disturbing force *F* acts on the GMA, and only the drive current *i* or the drive voltage *u* in the coil acts on the GMA. Then, the equivalent circuit can be expressed as inductance $L_{d}$ and resistance $R_{T}$, and the electrical properties are determined by the specifications of the coil and the material. In the dynamic case, when the external disturbing force acts on the GMA, the magnetization intensity will change with the permeability, and then, the magnetic induction intensity variation ▵B is produced. In the end, the series of changes results in the self-sensing voltage $u_{m}$.

In this paper, $L_{d}$ is approximately equal to 50 μH acordding to Equation (6) and i(t) is milliampere level. However, $u_{m}$ is millivolt level without amplification. So, part of F(t) caused by $L_{d}i$ is far less than that caused by $u_{m}$. According to $L_{d}i$ in Equation (7) ignored or not, the two cases are discussed in this paper, respectively. When $L_{d}i$ is ignored and only the factor $u_{m}$ is taken into account, the expression can be shown in Equation (8).

(8) $$F\left(t\right)=\frac{A_{r}}{A_{coil}Nd_{33}}\int u_{m}dt$$

If $L_{d}i$ is not ignored, i(t) can be estimated by $u_{m}$ and circuit impedance *Z*. Transfering Equation (7) from time domain to the Laplace domain, we can get Equation (9).

(9) $$F\left(s\right)=\frac{A_{r}}{A_{coil}Nd_{33}}\left(\frac{u_{m}}{s}-\frac{L_{d}}{Z}u_{m}\right)$$

**Figure 3:**
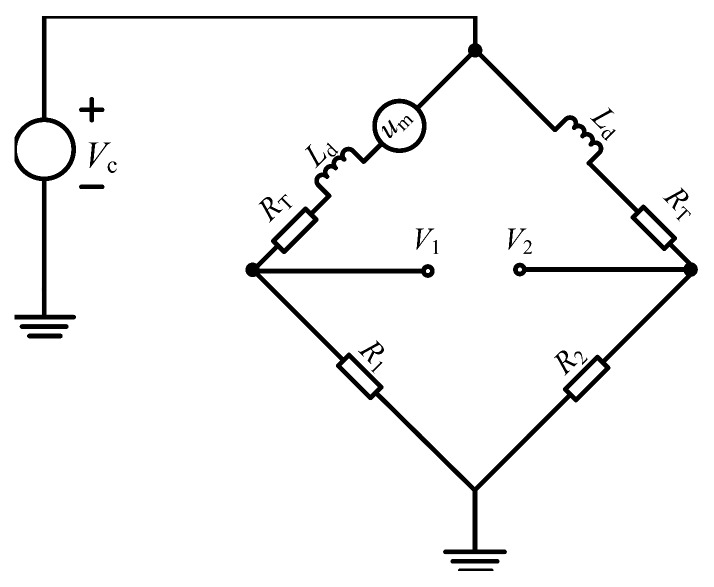
Principle diagram of the Wheatstone bridge circuit.

The conventional method using the Wheatstone bridge [10,14] for the extraction of the induced voltage is shown in Figure 3. When $R_{1}=R_{2}=R$, we can get the following equation in Laplace transform domain

(10) $$▵V=V_{1}\left(s\right)-V_{2}\left(s\right)=\frac{R}{R+R_{T}+L_{d}s}U_{m}\left(s\right)$$

From Equation (10), the bridge circuit is equivalent to a first-order low-pass filter for the bridge arm of the GMA-equivalent impedance model containing inductance. So, the GMA self-sensor work bandwidth is limited.

**Figure 4:**
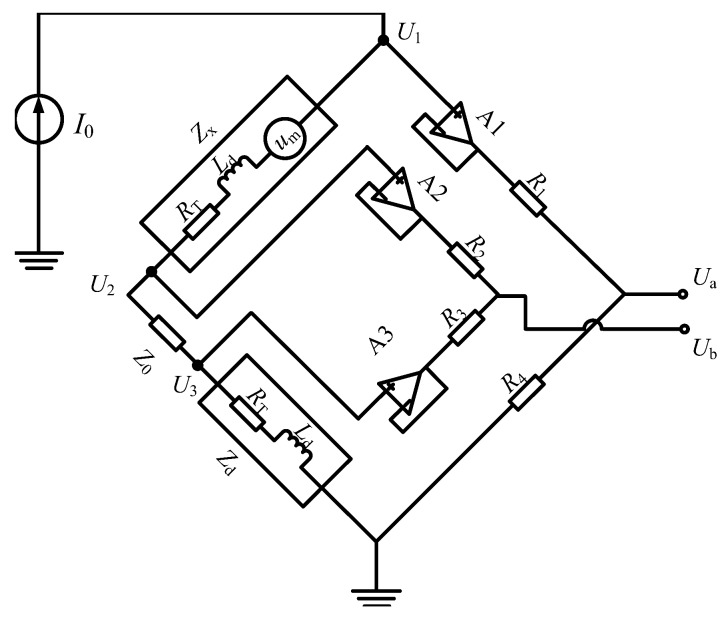
Principle diagram of the active Kelvin bridge circuit.

To solve the problem and realize linear extraction of the induced voltage, an active Kelvin bridge extraction method is discussed. On the one hand, an active Kelvin bridge with strong common mode rejection capability [21] is introduced into the extraction method. On the other hand, a reference actuator from the same batch products is adopted to balance the circuit bridge. The GMA and its reference actuator have similar impedance characteristics and the difference in the experimental measured values is in the milliohm level. The circuit principle diagram is given in Figure 4.

From the Laplace transform domain analysis of the above circuit, we obtain

(11) $$U_{1}\left(s\right)=U_{m}\left(s\right)+I_{0}\left(Z_{x}+Z_{0}+Z_{d}\right)$$

(12) $$U_{2}\left(s\right)=I_{0}\left(Z_{0}+Z_{d}\right)$$

(13) $$U_{3}\left(s\right)=I_{0}Z_{d}$$

(14) $$U_{a}\left(s\right)=\frac{R_{4}}{R_{1}+R_{4}}U_{1}\left(s\right)$$

(15) $$U_{b}\left(s\right)=\frac{R_{3}}{R_{2}+R_{3}}\left[U_{2}\left(s\right)-U_{3}\left(s\right)\right]+U_{3}\left(s\right)$$

Order $R_{1}=R_{2}=R_{3}=R_{4}$, thus

(16) $$▵U=U_{a}\left(s\right)-U_{b}\left(s\right)=\frac{1}{2}\left[U_{m}\left(s\right)+I_{0}\left(Z_{x}-Z_{d}\right)\right]$$

This means

(17) $$U_{m}\left(s\right)=2▵U-I_{0}\left(Z_{x}-Z_{d}\right)$$

Acorrding to Equation (16), the output voltage ▵u will be proportional to $u_{m}\left(t\right)$. The induced voltage is extracted linear, and it is not related with the frequency compared to the Wheatstone bridge method. This extraction method can be used over a broad bandwidth.

## 4. Experiment

**Figure 5:**
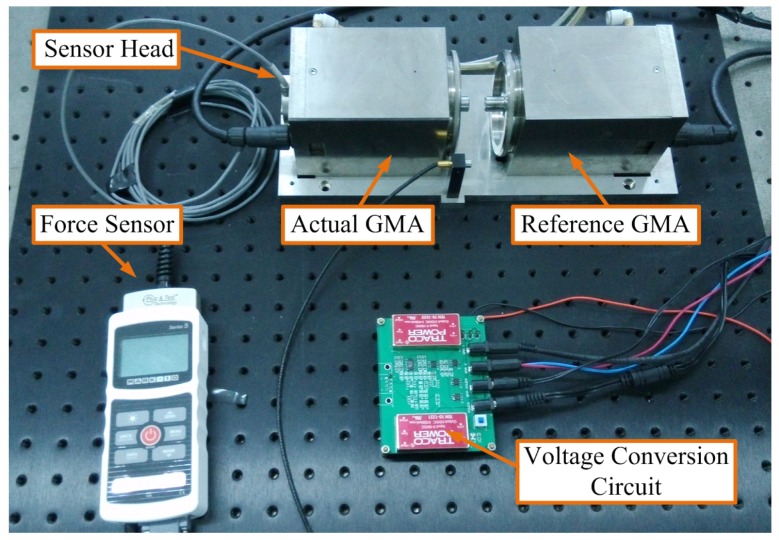
Photograph of experimental apparatus.

The experimental apparatus is shown in Figure 5. Two of the same type of GMA actuators having the same batch of GMM rods with the dimensions ϕ10×33mm${}^{3}$ installed are referred to as “actual GMA” and “reference GMA”, respectively. Actual GMA is selected for the output actuator with external disturbing force, and the reference GMA is selected as a reference actuator without the external disturbing force. Their respective static impedances measured by a multimeter Agilent 3458A are $R_{1}$ = 2.49 Ω and $R_{2}$ = 2.52 Ω, namely $Z_{x}-Z_{d}$ = 30 mΩ. $Z_{0}$ = 10 Ω. The external disturbing force directly acted on the output end of the actual GMA, and a standard force sensor is used to measure the disturbing force as a calibration for the self-sensing force.

The pre-bias test conditions are a preloading force of 143 N and a pre-bias magnetic field of 24 mT. The maximum output frequency of the MARK-10 I5 series sensor as the standard force sensor is 250 Hz, and its measuring sensitivity is ±0.5 N. The drive current is 0–0.01 A.

While GMA works under dynamic case, *i.e.*, the disturbing force *F* acts at the output end of the actual GMA through the standard force sensor, and the circuit board measurement of output voltage is ▵u; then, the self-sensing voltage $u_{m}$ is equal to 2▵u. According to Equation (8), the disturbing force *F* is integral of the voltage 2▵u, which means the relationship between force *F* and the integral of the voltage 2▵u is linear. Therefore, linear fitting can be done between the disturbing force *F* measured by the force sensor and the integral of the measured voltage $u_{m}$ in data processing.

Similarly, according to Equation (9), binary linear regression can be done to analyze the linear relationship between the three factors the disturbing force, the measured voltage and its intergral. The drive current selected in this experiment is 0 and 0.01 A, respectively.

**Figure 6:**
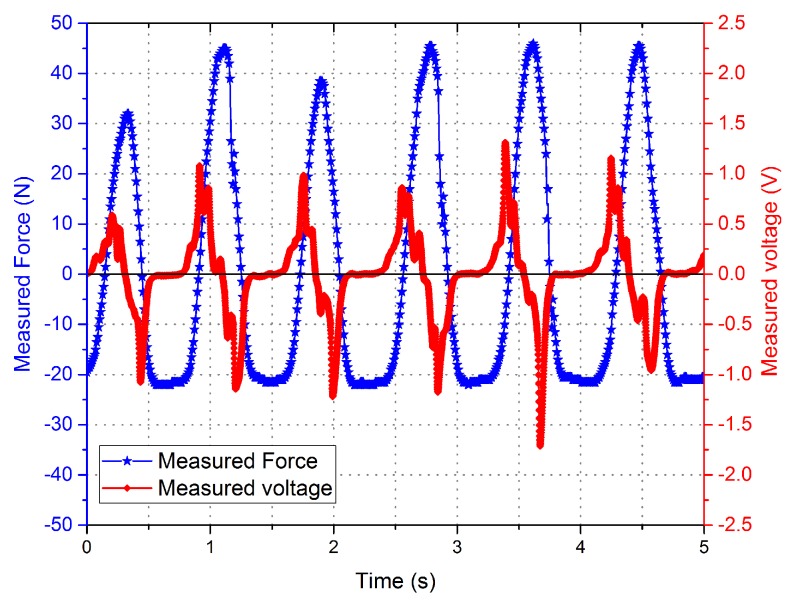
Measured force and the measured voltage curves with drive current = 0 A.

**Figure 7:**
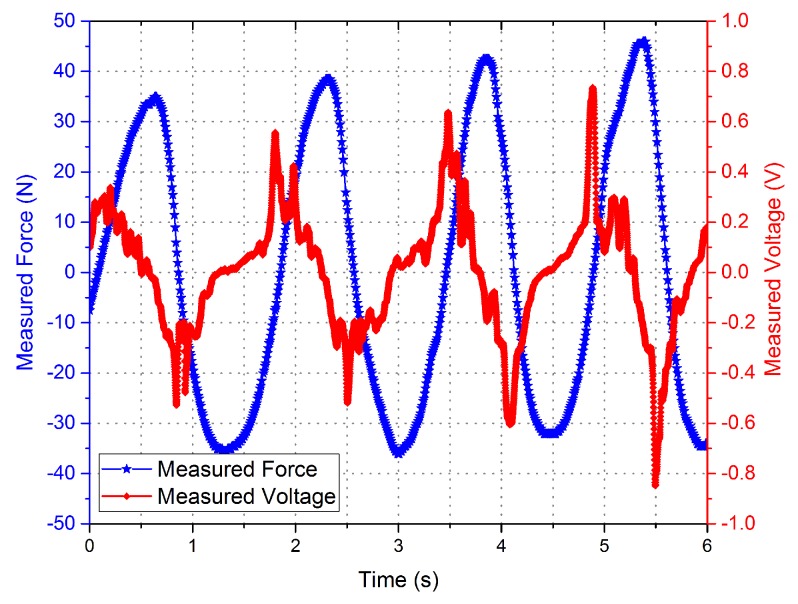
Measured force and the measured voltage curves with drive current = 0.01 A.

**Figure 8:**
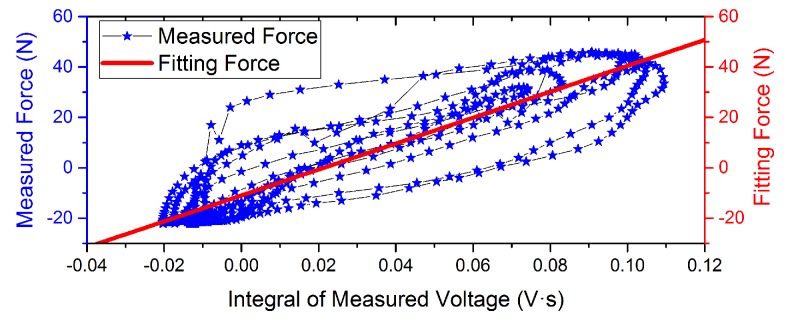
Fitting curves of the measured force and integral of voltage with drive current = 0 A.

**Figure 9:**
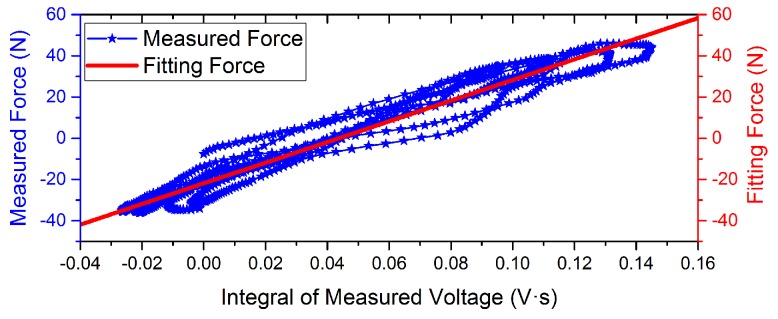
Fitting curves of the measured force and integral of voltage with drive current = 0.01 A.

Experimental measurement data with different driving current is shown in Figure 6 and Figure 7. The curves indicate that the phase of the voltage curve approximate 90${}^{\circ }$ ahead of the force curve. The fitting curve of the *F*-integral ▵u is shown in Figure 8 and Figure 9, and the fitting line symmetrical distribution in the middle of the measured curve can explain the probability distribution characteristics between them. The mathematical relationship is shown as follows:

(18) $$F\left(t\right)=k\int_{0}^{t}▵udt-b$$

In order to get the linear relation, the second part on the left side of Equation (9) should be simplified. According to the experimental conditions, the simplify process is shown as the follows:

(19) $$\frac{L_{d}}{Z}u_{m}=\frac{L_{d}}{2Z_{x}+Z_{0}}u_{m}=\frac{L_{d}}{2L_{d}s+2R_{T}+Z_{0}}u_{m}=\frac{1}{2s+\frac{2R_{T}+Z_{0}}{L_{d}}}u_{m}$$

The value of $\frac{2R_{T}+Z_{0}}{L_{d}}$ approximate 3 $\times \;10^{7}$ with the $L_{d}$ = 50 μH. So, $\frac{L_{d}}{Z}$ can be considerated as a first-order low-pass filter with a small gain. In other words, the filter gain can be simplified as a constant in low frequency range. So, the mathematical relationship of binary linear regression is as follows:

(20) $$F\left(t\right)=k\int_{0}^{t}▵udt-k^{\prime }▵u-b$$

According to the mathematical positive proportion relationship between them, the slope rate *k* of the straight line indicates that the proportion coefficient of the *F* relative to the integral voltage ▵u is equal to 514.64 N/(mV·s), and the change in intercept *b* with the input force indicates that the measurement offset is equal to 10.98 N with drive current = 0 A. When drive current = 0.01 A, *k* is equal to 501.24 N/(mV·s) and *b* is equal to 21.83 N. Moreover, the linearity with drive current = 0.01 A is better than static conditions. This results may be due to the common-mode rejection ability of the active Kelvin bridge. In the end, the parameters of regression analysis results are shown in Table 1. The correlation coefficient *r* decreased with consideration of the direct effect of induce voltage to the disturbing force. The self-sensing accuracy is improved.

**Figure 10:**
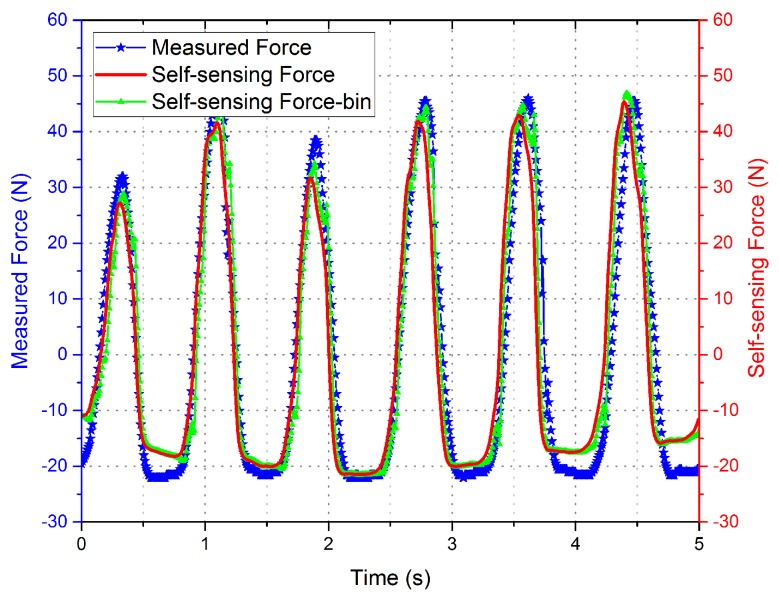
Comparison of the self-sensing and measured force curves with drive current = 0 A.

**Figure 11:**
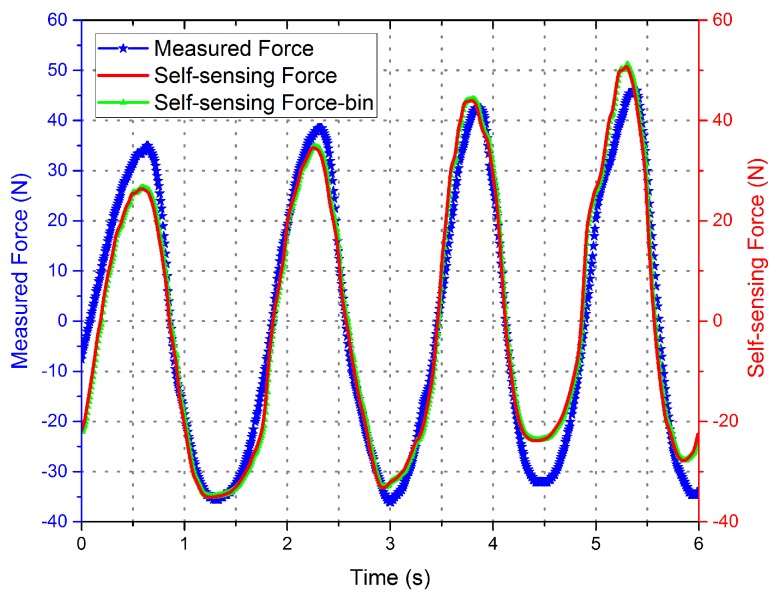
Comparison of the self-sensing and measured force curves with drive current = 0.01 A.

The curve of the self-sensing force calculated by the self-sensing voltage and the fitting relation Equation (18) is shown in Figure 10 and Figure 11, with the disturbing force measured by the force sensor shown for contrast. The data of the self-sensing force are close to those of the measured force at most positions. The experiment proved the availability of the linear extraction method for induced voltage. Although the curve of the binary regression analysis is more close to the measured curve, the effectiveness improvement between the binary regression and monadic regression are not obvious compared to the error of self-sensing.

The GMA self-sensing sensitivity coefficient *S* is defined as the integral of self-sensing voltage caused by disturbing force as follows.

(21) $$S=\frac{\int u_{m}dt}{F}=\frac{1}{k}$$

Experimental results show that the GMA self-sensing sensitivity coefficient is better than 2.0 (mV·s)/N. According to the results of the fitting curves, the sensitivity coefficient *S* equal to 1.94 (mV·s)/N and 2.00 (mV·s)/N with the slope rate *k* equal to 514.64 N/(mV·s) and 501.24 N/(mV·s), respectively.

The data of the self-sensing force are close to those of the measured force at most positions, but obvious errors caused by the nonlinearity of the prestressed spring appear in the peak and trough of the curve. The error is affected by three key factors, namely the nonlinearity and hysteresis of the GMA itself, the measurement accuracy of the standard force sensor, and the measurement error of the self-sensing circuit.

## 5. Conclusions

In this paper, a method for solving the nonlinear problems in GMA self-sensing signal extraction has been demonstrated. Dynamic equivalent circuit model of GMA was analyzed with the concept of an active Kelvin bridge. Induced voltage was extracted with no distortion and no loss of the high frequency information due to the strong common-mode rejection ability by the active device. The experimental results reveal that the Kelvin bridge circuit extracting the self-sensing signal is more effective than the conventional Wheatstone bridge. Further work in the GMA self-sensing will include analysis and nonlinear correction of the permeability through experimental measurements in order to compensate for the self-sensing performance of the device.

## Figures and Tables

**Table 1:** Parameters of regression analysis.

Drive Current	*F*-Integral ▵u			*F*-Integral ▵u − ▵u			
	*k* (N/(mV·s))	*b* (N)	*r*	*k* (N/(mV·s))	$k^{\prime }$ (N/(mV))	*b* (N)	*r*
0 A	514.64	10.98	0.9273	515.36	14.81	11.00	0.9630
0.01 A	501.24	21.83	0.9716	501.47	6.60	21.71	0.9771

## Abbreviations

The following abbreviations are used in this manuscript:

GMA: Giant Magnetostrictive Actuator
GMM: Giant Magnetostrictive Material
